# Health, Wellbeing, and Social Interaction: An International and Demographic Analysis of Perceived Life Changes and the Positives and Negatives of the COVID-19 Lockdown

**DOI:** 10.5964/ejop.7751

**Published:** 2023-05-31

**Authors:** Jennifer Murray, Mandeep K. Dhami, Kirstie McClatchey, Leonardo Weiss-Cohen, Peter Ayton

**Affiliations:** 1 School of Health and Social Care, Edinburgh Napier University, Edinburgh, United Kingdom; 2 Department of Psychology, Middlesex University, London, United Kingdom; 3 Usher Institute, University of Edinburgh, Edinburgh, United Kingdom; 4 Department of Psychology, Kingston University London, London, United Kingdom; 5 Leeds University Business School, University of Leeds, Leeds, United Kingdom; Università Cattolica del Sacro Cuore, Milan, Italy

**Keywords:** COVID-19, lockdown, social interaction, wellbeing, perceptions of change

## Abstract

Research suggests that people’s experiences of COVID-19 lockdowns have been detrimental to their lives and wellbeing. The current research compared the experiences and perceptions on health, wellbeing and social interaction of 300 UK adults and 450 adults in California. Individuals reported whether aspects of their life had changed for the better, worse, or not at all during lockdown in April 2020, and what the “best” and “worst” things about lockdown were. There were more similarities than differences in the regional comparison of perceptions of changes in specific aspects of ‘health and wellbeing’ and ‘social interaction’. Both regions reported the same number and nature of best and worst things about lockdown. Overarching themes of ‘health, self and wellbeing’, ‘being with others’, and ‘concerns with daily living’ were identified. Although reports of life changes and the positives and negatives of lockdown were similar across different demographic groups, some differences were present by age, sex, relationship, and family-status. Incorporating knowledge of unified and positive experiences of lockdown can be useful in informing future lockdown restrictions and supporting the population when restrictions are lifted.

The United States of America (US) and the United Kingdom (UK) both confirmed their first COVID-19 cases at the end of January 2020 (World Health Organization, 2020a, 2020b) and global efforts to slow the spread of COVID-19 have been in place to varying degrees since January 2020. In California, US, a mandatory Stay-at-Home Order was announced on 19th March 2020 (California All, 2020), with the UK announcing a similar approach only days later, on 23rd March (Cabinet Office, 2020). By April 2020, over a fifth of the world’s population was placed under similar lockdowns (Gilbert, 2020). The severity of the restrictions placed on people under these lockdowns have varied, with some governments requiring people to stay indoors at all times with the exception of keyworkers and emergencies, while others requested that citizens restrict movement to only essential journeys and food shopping. Enforcement also varied, from police warnings and fines to imprisonment (e.g., Executive Department State of California, 2020; UK Government, 2020). A key feature of lockdowns across countries was the closing of schools, childcare facilities, and play parks, closed workplaces with people being requested to work from home where possible, going into furlough, or losing their employment altogether, closure of places of worship, closing of non-essential businesses such as social spaces, restaurants, gyms, and the closure of the majority of retail establishments.

The current research presents an international comparison of people’s experiences during this initial period of lockdown, comparing responses from the US (California) and the UK. The research specifically asked people to self-report what they felt were the best and worst things about lockdown for them, and their perceptions of changes in their life since lockdown. Through comparing data across the two regions, we aim to identify whether there are region-specific differences in people’s reported lockdown experiences or whether the experience of lockdown has been relatively similar. This exploration adds important insights on people’s social and psychological experiences of lockdown, which was noted as a gap in the literature by Bavel et al. (2020), and from a broader perspective this may further inform us about appropriate policy responses to ongoing social isolation and possible future lockdowns, including supporting people who are isolated both during and possibly outwith times of pandemic restrictions.

Throughout this paper we discuss health, which is a complex construct. We adopt the Constitution of the World Health Organization (1946) definition of health, which includes physical, mental, and social wellbeing, viewing health in a holistic way: “Health is a state of complete physical, mental and social well-being and not merely the absence of disease or infirmity.”

## Research on COVID-19 Lockdown Experiences

Within the literature on COVID-19 lockdown experiences, important findings around psychological, emotional and social wellbeing have been reported. Among these findings, themes of loneliness, isolation, anxiety and other psychological effects have been reported. We will summarise key findings from the literature on these broad topics first. We will also discuss research which identified issues around perceived and lived experiences of social isolation, lack of freedom and the impact on physical wellbeing. Following this section, we will then discuss in more depth research which sought to compare different groups’ experiences of the COVID-19 lockdown, with a focus on the relevant themes already mentioned, with a particular focus on mental health and wellbeing.

Loneliness has been discussed as a growing problem which leads to negative effects on mental health, wellbeing, anxiety (Cacioppo & Cacioppo, 2018), and on cognitive function (Shankar et al., 2013), long before the lockdown measures applied in recent years. Unsurprisingly, lockdown has had a significant detrimental impact on wellbeing, with self-report research identifying increased anxiety and poorer mental health in lockdown (e.g., Ali et al., 2020; Aymerich-Franch, 2020; Odriozola-González et al., 2020; Park et al., 2020; Roy et al., 2020; Satici et al., 2022; Sharma & Subramanyam, 2020; Stieger et al., 2021; Vijayaraghavan & Singhal, 2020; White & Van Der Boor, 2020). Research by the Office for National Statistics (ONS) in the UK found that between early April and May 2020, around a third of those surveyed reported that their wellbeing had been negatively affected through feelings of loneliness in the past seven days (ONS, 2020a). Further survey research from the UK exploring the period immediately post-lockdown, found that almost one-third of respondents reported loneliness, with a higher risk of loneliness for younger age groups, and those separated or divorced (Groarke et al., 2020). These researchers found that having higher levels of social support or being married or co-habiting acted as protective factors against loneliness.

Evidence from New Zealand also suggested that approximately a third of survey respondents were experiencing moderate to severe psychological distress during lockdown (Every-Palmer et al., 2020), again with poorer outcomes seen in younger people, which echoes the wider research highlighting that young people experienced higher stress, anxiety and/or negative affect than older people during lockdown (Aymerich-Franch, 2020; Park et al., 2020; Vijayaraghavan & Singhal, 2020).

Interestingly, of the respondents in Every-Palmer et al.’s (2020) study, two-thirds were able to highlight a number of positives of lockdown, which included enjoying working from home, spending more time with family, and having a quieter, less polluted environment. Similarly, in the UK, Williams et al. (2020) found that participants were able to draw positives from lockdown. This included having more time with children and bringing families together. However, the authors noted that participants who explicitly discussed positives to lockdown were from higher socioeconomic backgrounds and tended to live in more rural areas. Despite socioeconomic status being stated by these authors as important within interpreting the findings, no specific, objective measurement nor definition of socioeconomic status was actually reported in the study. As socioeconomic status is a complex construct, this finding is somewhat ambiguous in an objective sense. The authors do, however, report on *standard occupational classification*, which, as defined by the ONS (2020b), classifies jobs in terms of their skill content and skill level. In the study the majority of participants were employed in a professional or skilled role or were in full time education. In addition, and despite some positives being highlighted, the main findings of Williams et al.’s (2020) study was that participants felt depressed and anxious as a result of lockdown, with some likening the experience to prison (see also Dhami et al., 2020).

This impact on freedom has been mirrored in other findings, with 58% of a UK sample indicating ‘lack of freedom’ as the most common experience of lockdown (ONS, 2020c). This is despite 91% of the sample having left their homes during the same time period, with 49% leaving to visit a park/green space, and 40% still travelling for employment (ONS, 2020c). These findings are similar to those in Italy, where lack of freedom was also found to be the most common negative experience of lockdown (Barari et al., 2020).

As well as the impact on mental wellbeing, lockdown has had an impact physical wellbeing. Williams et al. (2020) found that participants spoke of a loss of motivation to perform basic everyday tasks. Additionally, research has found that physical activity decreased during lockdown, as did consumption of fruit and vegetables (Naughton et al., 2021). Furthermore, alcohol consumption increased during the pandemic in both UK and US samples (Naughton et al., 2021; Pollard et al., 2020).

Finally, lockdowns have also affected day-to-day living. Research has highlighted that the inability to go to work, the significant restructuring of work patterns, including balancing home working with home schooling, combined with worry over the virus itself, has left people feeling ‘overwhelmed’ or ‘scared’ (Williams et al., 2020). This change in work-life balance has been particularly marked for women, with females being more likely to state that their work-life balance deteriorated during lockdown (Ashencaen Crabtree et al., 2021).

## Empirical Comparisons of Lockdown Experiences

The growing body of research investigating the effects of the pandemic and lockdowns has mainly focused on negative experiences and has been situated within the initial lockdown period in Spring 2020. This research is important as it not only mapped people’s initial adverse experiences of lockdown but may also help to inform how people will experience and cope with the ongoing lockdowns and varying degrees of stay-at-home orders within the ongoing pandemic situation. However, little research has explored positive experiences of lockdown and much research to-date has focused on measuring people’s experiences at a single point in time, investigating a narrow range of variables, and have focused on single-country level data. There are some exceptions however, where researchers either investigated people’s perceptions of changes over the initial lockdown period or measured changes in a longitudinal manner, although these studies do not explicitly measure positive experiences.

Sibley et al. (2020), for instance, compared the mental and physical health and subjective wellbeing of a matched sample of adults in New Zealand a few months before the lockdown and during the first 18 days of lockdown in addition to conducting within-subjects analyses using data from the same participants a year earlier. Both sets of analyses showed an effect of lockdown (e.g., increased mental distress and sense of community after lockdown, but decreased fatigue), although this was only the case for a minority of the measures. Participants were found to be generally resilient either compared to matched others or to themselves. While this research had a robust methodology, it is clear retrospectively that New Zealand took a different—more successful—approach to the COVID-19 pandemic situation than many other countries in the world, having had a more effective reduction in cases (World Health Organization, 2020c). The generalisability of the findings of this research to other countries may, therefore, be reduced due to the differing political and public health messaging approaches taken in New Zealand in comparison to the majority of other countries during the first lockdown, with proactive measures such as coordinated public health information campaigns and the banning of non-citizen arrivals to some regions of New Zealand beginning on February 2nd, 2020; 24 days before the country’s first diagnosed case (Baker et al., 2020).

Looking to longitudinal and comparative research, Pierce et al. (2020) investigated changes in people’s pre-lockdown and in-lockdown mental health among over 17,000 UK adults. The study used data collected pre-lockdown in the UK Household Longitudinal Study (UKHLS) and a follow up survey in April 2020, using the General Health Questionnaire (GHQ-12) to assess mental health. Deterioration in mental health was most present in women, people aged 18–24 years, and with people living with young children. This decrease in mental health mirrors Sibley et al.’s (2020) findings, as do those of Niedzwiedz et al. (2021), who conducted a longitudinal (four-wave) survey between 2015 and 2020 in the UK using the same measures as Pierce et al. (2020). In addition to young people and women being most affected, this latter study also found that people from an Asian background and those who were educated to degree level were at a higher risk of poorer mental health one month into lockdown. Niedzwiedz et al. (2021) classified degree level as the highest educational attainment within their coding, though did not expand on the type of degree attained by participants.

In contrast, O’Connor et al. (2021) carried out a three-wave survey of over 3,000 adults during the first UK lockdown examining mental health and wellbeing. While they found that suicidal ideation increased over the lockdown time period, anxiety, feelings of defeat and entrapment decreased, and symptoms of depression and loneliness remained steady. This study also identified reports of positive changes in wellbeing among some participants. However, women were once again identified to be at higher risk of poorer mental health during lockdown. Thus, despite the clearly negative impact on some aspects of mental health and wellbeing, the effect of time during lockdown on changes in people’s experiences of mental health and wellbeing presents a more complex picture, demonstrating the need for ongoing research and research which looks at real and perceived changes in experience.

Dhami et al. (2020) present one of the few studies comparing people’s experiences of lockdown against non-COVID lockdown samples. They compared 750 UK and Californian adults’ lockdown experiences with 573 first-time prisoners’ experiences of pre-pandemic imprisonment to determine whether any parallels existed between the two forms of confinement. The analyses were conducted per region (with the prison samples from each region). There were surprising differences among the lockdown and prison samples. People in lockdown engaged in a fewer variety of daily (life enriching and coping) activities and felt more hopeless than did first-time prisoners.

It is clear that lockdowns in response to the COVID-19 pandemic have had a significant impact on people’s lives globally (Bavli et al., 2020), and there are some indications that experiences of lockdown may have unified features across countries. Therefore, the present study explores whether the responses to lockdown are country-specific, or if there is a more shared experience of lockdown. A comparative analysis of what people feel are the best and worst things about lockdown was carried out across a UK and US (California) sample. In addition, to address the issue of single time-point data being a feature in the majority of research, the current research also investigated people’s perceptions of change over the lockdown and asked whether they perceived aspects of their lives to have gotten better, worse or remained the same. Through the current investigation, a clearer picture of people’s self-reported best and worst experiences of lockdown and perceived changes in their lives will be presented, in addition to the international (UK, US) comparison.

## Method

### Design

An online survey was conducted in the UK and the US State of California. Data were collected in the UK from April 20th to 22nd and in California from April 24th to 28th. The lockdown in both regions began around the same time (i.e., March 23rd and 20th, 2020, respectively). Both regions are Anglophone but are culturally and politically different, thus allowing a comparison of two regions which shared a similar nature and timing experience of lockdown but which are not identical in their cultures. The current paper reports the qualitative data collected within the survey and data relating to a series of change variables. The qualitative data were collected through two open-ended questions asking the participants’ perceptions of the “best” and “worst” things about lockdown. Data were grouped using an inductive thematic analysis approach (Braun & Clarke, 2006) and coded to allow quantitative comparisons between the different groups. The change variables (see Appendix 1 in the Supplementary Materials) asked people nine questions about their perceived changes in behaviours since the lockdown began compared to beforehand using 7-point ordinal scales for most questions and one nominal list of behaviours which had been stopped during the course of lockdown. Specific Independent and Dependent Variables are detailed within the results sections for each statistical test run. A pilot was not carried out specifically for this research; however, the study design was based on successful research and survey design employed in past research (i.e., Souza & Dhami, 2010). The study design allowed a rich collection of qualitative and quantitative data on the topic to be collected in such a way as to allow detailed comparisons, as presented in the 'Results' section.

### Participants

Three hundred adults in lockdown in the UK and 450 in California responded to the survey. Participants were recruited via convenience sampling via online advert for the study. Due to the lockdown situation, online research was appropriate and sensible given the lack of face to face contact possible and to allow as broad a sample to be collected from the general population as possible. Table 1 presents the demographic characteristics of the two samples.

**Table 1:** Demographic Characteristics of UK and Californian Samples

	UK (*N* = 300)		California (*N* = 450)	
Characteristic	*M* (*SD*)	%	*M* (*SD*)	%
Female		63.8		48.8
Ethnicity–BAME		13.0		56.9
Disability		5.7		0.0
Up to high school only		75.3		14.4
Unemployed		12.8		15.8
Income assistance		11.4		9.1
Not in relationship		65.0		60.6
Shared household		90.7		84.9
Age	33.07 (11.14)		30.62 (11.22)	
Number of children	0.69 (1.01)		0.36 (0.88)	
Days in lockdown	30.17 (7.79)		37.66 (9.63)	

*Note*. BAME = Black, Asian and Minority Ethnic.

### Materials

The survey, called “Life in Lockdown,” comprised six sections (i.e., ‘Your Life in Lockdown’, ‘Socializing with Others’, ‘Your Health’, ‘Rules of Lockdown’, ‘Your Experiences of COVID-19’, and ‘You and Your Life Before Lockdown’). The current paper reports the qualitative data collected within the survey and quantitative data relating to a series of “change” variables. The full survey can be found in the Supplementary Materials and analyses of other survey items are reported in Dhami et al. (2020). The survey included several quantitative items asking participants to report changes that had occurred during lockdown in comparison to before lockdown. These items can be grouped into those asking about health and wellbeing, and those asking about social interactions, including both contact with family/friends in- and outside of participants’ households. The items and response scales are provided in Appendix 1 in the Supplementary Materials.

The survey also contained two open-ended items adapted from Souza and Dhami (2010). These were: “What is the [best/worst] thing about being in lockdown”. Participants could choose to answer one, both, or neither question. Responses were collected single free-text entry boxes with no character limit for each question.

### Procedure

Participants in the lockdown samples were recruited online using Prolific Academic. Only individuals who reported being fluent in English and currently residing in the UK or California were allowed to participate. Upon completion, each participant was paid £2 (or its USD equivalent). Participants could skip questions but could not go back to change previous answers. Ethical approval for the research was granted by the Research Ethics Committee at City University, London.

## Results

### Perceptions of Life Change Since Lockdown

Table 2 presents the means and standard deviations of the change variables by Sample. We conducted three sets of analyses to determine if participants’ reports of the changes that occurred to their lives after the first COVID-19 lockdown were similar or different across the two samples, taking into account any differences in the demographic characteristics of the samples.

**Table 2:** Means and Standard Deviations of Dependent Variables by Sample

	UK		California	
Dependent Variable	*M* (*SD*)	%	*M* (*SD*)	%
*Health & wellbeing*				
Given up habits	.40 (.58)		.66 (.73)	
Food quality^+^	.03 (1.26)		-.07 (1.20)	
General health^+^	-.11 (.93)		-.11 (.98)	
Self-image^+^	-.04 (1.08)		-.07 (1.01)	
Self-improvement		37.0		38.1
*Social interaction*
Mix with household^+^	.89 (1.34)		.72 (1.75)	
Contact outside^+^	.04 (1.74)		-.10 (1.64)	
Social life^+^	-1.29 (1.25)		-1.07 (1.24)	
Treatment by others^+^	.20 (.69)		.15 (.69)	

*Note*. ^+^These variables were measured on a –3 to +3 scale with a 0 midpoint.

First, a MANCOVA was used to determine the effect of Sample (UK or California) on six of the measures of change (i.e., food quality, general health, self-image, social life, contact with family/friends outside household, and treatment by family/friends). The following demographic factors were entered as covariates: age, gender (male or female), ethnicity (BAME or White), education level (up to high school only or beyond), employment status (unemployed or employed), income assistance (no or yes), relationship status (alone or in relationship), number of children, shared household (no or yes), and number of days in lockdown. The absolute correlations among the six variables entered in the MANOVA ranged from *r* = .001 to .37. There was no significant multivariate effect of Sample on these variables, *F*(6, 703) = 1.18, *p* = .314, $\eta_{p}^{2}$ = .010, and no univariate effects. None of the demographics were significantly associated with the six measures of change.

An ANCOVA was then used to examine the effect of Sample on change in the mixing with others in the same household. This question was only shown to participants who indicated sharing a household, and therefore covered a smaller subset of participants. The analysis had to exclude the shared household variable, which was equal to ‘Yes’ for all participants. It was found that after controlling for significant effects of relationship status (*p* = .002) and number of children (*p* = .028), there was no significant effect of Sample on amount of mixing with others in household *F*(1, 622) = 0.58, *p* = .447, $\eta_{p}^{2}$ = .001. Individuals in a relationship demonstrated a significant increase in the amount they mixed with others in their household since lockdown began (*M* = 1.10, *SD* = 1.72) than those not in a relationship (*M* = .59, *SD* = 1.66), *t*(646) = -3.77, *p* < .001. The number of children an individual said they had was significantly positively associated with a change in the amount of mixing with others in their household since lockdown began, *r* = .14, *p* = .001.

Finally, logistic regression analysis was used to measure the predictive validity of Sample on whether or not participants began a new hobby or self-help, controlling for the demographic factors. This revealed that the only significant predictor of self-improvement was gender, *B* = .37 (*SE* = .16), Wald (*df* = 1) = 5.26, *p* = .022. The odds of males beginning self-improvement was 1.45 times more likely than females.

### Best and Worst Things About Lockdown

Analysis of the two qualitative items occurred in four steps: (1) inductive development of coding categories (Braun & Clarke, 2006); (2) coding of data into these categories; (3) coder reliability check; and (4) comparative analysis. First, the data corpus was read and re-read by one author (JM) and an initial set of coding categories was developed. Following discussions between two authors (JM and MKD) and a process of grouping and re-grouping the data and categories, 18 distinct and meaningful themes for the “best” item and 20 themes for the “worst” item were agreed. These were then grouped into three themes for each question. Where a participant’s response aligned to more than one category/theme, it was coded within all applicable categories and themes. Then, a reliability analysis was performed using a coder with no involvement in the study design, administration, or analysis (KMcC). The results of the reliability analysis are provided in Appendix 2 in the Supplementary Materials. The coding was discussed (JM and KMcC) until 100% agreement was reached. Finally, responses from the UK and Californian samples were compared using a combination of independent samples t-tests, Chi-square tests of independence and logistic regression analyses. Again, these comparative analyses took account of any demographic differences between the two samples.

The numbers of best and worst things about lockdown expressed by participants were compared across the two samples using two independent samples t-tests (see Table 3). There were no significant differences in the numbers of best and worst things about lockdown across the UK and Californian samples (Best thing: *t*[747] = 0.01, *p* = .995, Worst thing: *t*[688.08] = -1.21, *p* = .228).

**Table 3:** Mean (SD) Number of Best and Worst Things about Lockdown by Regional Sample

	UK (*N* = 300)		California (*N* = 449)	
Value	*M*	*SD*	*M*	*SD*
Best	1.36	0.66	1.36	0.65
Worst	1.33	0.60	1.39	0.67

Appendix 3, in the Supplementary Materials, presents the percentage and number of times that responses regarding the best and worst things about being in lockdown were coded into general themes and specific categories within the whole dataset and also within each sample. When a participant’s response to the question aligned within a particular theme this was then coded as being present for that participant. Table 4 presents the participant level data. Comparisons for each of the three overall themes for the best (i.e., ‘health, self, and wellbeing’, ‘being with other people’, ‘daily living concerns’) and worst thing responses (i.e., ‘health, self, and wellbeing’, ‘being with other people’, ‘daily living concerns’) were conducted using chi-square tests of independence. There were no significant associations between Sample and perceptions that the ‘Best’ thing about lockdown were improvements in ‘health, self, and wellbeing’, χ^2^(1, *N* = 749) = .26, *p* = .607, beliefs that ‘being with other people’, χ^2^(1, *N* = 749) = .46, *p* = .497, nor between improvements in ‘daily living concerns’, χ^2^(1, *N* = 749) = .03, *p* = .871.

**Table 4:** Percentage of Participants by Regional Sample Whose Responses for Best and Worst Thing About Lockdown Were Coded Into Broad Themes

Themes	All (*N* = 749)	UK (*N* = 300)	Californian (*N* = 449)
***Best Thing about Lockdown***			
Health, Self, and Wellbeing	38.5	37.3	39.2
Being with other people	32.6	34.0	31.6
Daily living concerns	37.0	37.3	36.7
***Worst Thing about Lockdown***			
Health, Self, and Wellbeing	26.8	25.0	28.1
Being with other people	41.5	44.7	39.4
Daily living concerns	53.4	49.0	56.3

*Note*. The *N* and percentages in Appendix 3, in the Supplementary Materials, and Table 4 differ due to the different nature of the data being presented. The data presented in this table represent the participant level responses rather than the total number of ‘data extracts’ within the themes across the entire data corpus.

There were also no significant associations between Sample and perceptions that the Worst thing about lockdown were reductions in ‘health, self, and wellbeing’, χ^2^(1, *N* = 749) = 0.86, *p* = .354, or beliefs around ‘being with other people’, χ^2^(1, *N* = 749) = 2.04, *p* = .153. The association between Sample and perceptions of ‘daily living concerns’ as being the worst thing about lockdown was statistically significant, χ^2^(1, *N* = 749) = 3.90, *p* = .048, with participants in California being slightly more likely to report such concerns than those in the UK (see Table 4).

Further chi-square tests were conducted to interrogate the association between Sample and each of the specific categories coded within the best and worst thing themes. For the ‘Best’ thing about lockdown, the only significant associations were those between Sample and: enjoying the outdoors and environment, χ^2^(1, *N* = 749) = 4.28, *p* = .039, with people in the UK being more likely to express this than people in California; not having to leave home, χ^2^(1, *N* = 749) = 6.02, *p* = .014, with people in California more likely to express this than people in the UK; and life being less hectic, χ^2^(1, *N* = 749) = 9.84, *p* = .002, with the UK sample expressing this more often. For the ‘Worst’ thing about lockdown, only the association between Sample and loneliness was significant, χ^2^(1, *N* = 749) = 4.52, *p* = .033, with people in California more likely to express this to be the worst thing about lockdown than people in the UK. The remaining non-significant chi-square analyses are available in Appendix 4, in the Supplementary Materials, in the Supplementary Materials.

Finally, logistic regression analyses were conducted to identify whether demographic characteristics could predict participants’ expression of the themes (i.e., ‘health, self, and wellbeing’, ‘being with others’, and ‘daily living concerns’) as the best or worst thing about lockdown. Within the ‘Best’ thing about lockdown responses, significant predictors for ‘health, self, and wellbeing’ were being in a relationship, *B* = -.49 (*SE* = .20), Wald (*df* = 1) = 6.30, *p* = .012, and having children, *B* = -.33 (*SE* = .12), Wald (*df* = 1) = 7.13, *p* = .01. Participants in a relationship and those with more children were more likely to view health, self and wellbeing as the best thing about lockdown. For ‘being with other people’, being in a relationship, *B* = .41 (*SE* = .20), Wald (*df* = 1) = 4.19, *p* = .041, and living in shared accommodation, *B* = -.65 (*SE* = .30), Wald (*df* = 1) = 4.79, *p* = .029, were significant predictors. Those in a relationship were more likely to express this as one of the best things about lockdown, but those living with others in shared accommodation were less likely to state this than people living alone. There were no significant predictors for ‘daily living concerns’ as one of the best things about being in lockdown.

Within the ‘Worst’ thing about lockdown responses, having a disability, *B* = 1.36 (*SE* = .56), Wald (*df* = 1) = 5.99, *p* = .014, was a significant predictor for expressing ‘health, self, and wellbeing’. For the ‘being with others’, age, *B* = -.03 (*SE* = .01), Wald (*df* = 1) = 8.20, *p* = .004, gender, *B* = .37 (*SE* = .16), Wald (*df* = 1) = 5.57, *p* = .018, and ethnicity, *B* = .40 (*S*E = .18), Wald (*df* = 1) = 4.97, *p* = .026, were all significant predictors. Younger people were less likely to consider this theme to be the worst thing about lockdown, while female and white participants were more likely to express this as one of the worst things about being in lockdown. Having children predicted ‘daily living concerns’ as the worst thing about lockdown, *B* = -.28 (*SE* = .11), Wald (*df* = 1) = 6.59, *p* = .010, with participants with more children being less likely to express this than those with fewer children.

## Discussion

The current research investigated whether there were common experiences of UK and Californian adult’s perceptions of life changes since lockdown, as well as their perceptions of the best and worst things about lockdown. Overall, we found more similarities than differences between the two regional samples. Thus, across these two regions, people appear to have shared experiences of lockdown, and in particular, similar to the findings of O’Connor et al. (2021), people do have positives experiences of lockdown; it is not a wholly negative experience.

The data collected reflects a snapshot in time, although participants were asked to report whether specific aspects of their lives had gotten better, worse or remained the same while in lockdown. We acknowledge that this does not replace the more robust method of pre- and post-lockdown data collection, however, the present findings regarding perceptions of life changes since lockdown do echo some of those reported in longitudinal studies.

When considering what people felt was the worst thing about lockdown, however, the current findings more clearly mirror those of the past longitudinal studies (Niedzwiedz et al., 2021; O’Connor et al., 2021; Pierce et al., 2020), with young people and females being more likely to express that aspects of being with others/not being with others was the worst thing about lockdown. Unlike the findings of Niedzwiedz et al. (2021), Pierce et al. (2020), and Sibley et al. (2020), the current research did not find that females significantly differed from males in terms of their perceptions of life changes but did find that males were more likely to begin self-improvement activities than females. It is unclear if this is due to females already engaging in such activities before lockdown, but if so, then lockdown means that males may benefit equally as females from a new hobby or self-help. Alternatively, some research suggests that females may be too burdened by household chores and childcare (Czymara et al., 2021; Yerkes et al., 2020) and so they may not have the opportunity to begin activities that can enrich them personally.

There was also a change in social interaction depending on demographics of the sample, with individuals in a relationship and those with more children showing a significant increase in the amount that they mixed with others in their household since lockdown began. The valence of the experiences of these two groups, however, differed. Findings regarding the best/worst thing about lockdown suggest that those in a relationship report aspects of improved health, self and wellbeing as the best thing about lockdown, whereas those with more children report that problems with daily living concerns/routine was the worst thing about lockdown. Their counterparts without children stated that improved health, self and wellbeing was the best thing about lockdown, and those not in a relationship and not in shared living situations valuing being with others most.

Considering the responses to the best and worst things about lockdown in more detail and what this means for people’s experiences of lockdown, spending time with others and being able to engage in routine or ‘normal’ activities are clearly central to a person’s positive or negative experience of lockdown. The most common best thing about being in lockdown was being able to spend more time with others, including family, friends and pets. This included spending time with people living in the same accommodation and spending time with people virtually via online platforms. The next most common response was having time to learn and to do things such as hobbies, education, and catching up on DIY projects. In parallel, the most commonly expressed worst thing about lockdown were being physically apart from others and not being able to do routine, everyday things and activities.

Although similar patterns or themes were observed for the best and worst thing about lockdown there were also observable differences. When considering the best thing about lockdown, individuals expressed a positive change in their perceptions of life, having more time for self, more sleep, rest and relaxation, a less hectic pace of life, less social pressure and appreciating the space and privacy of the lockdown experience. In contrast, with regard to the worst thing about lockdown, individuals expressed concerns over the uncontrollable passing of time, concerns and stress over the uncertainty of the situation, a lack of freedom, space, privacy and peace, the increased home pressures with home childcare and education in addition to home working, and having too much time with the family, in addition to experiences of negative psychological states such as deteriorating mental health, anger, frustration, boredom, anxiety, fear, and loneliness.

These patterns, echoed in the analysis of the change variables, indicate that there are two key types of experiences of lockdown. The first appears more positive, with a flexible approach to living, where finances, employment, and engaging (to a greater extent than before) in routine activities such as hobbies and projects are not negatively impacted. Here, individuals appear to have more time for themselves or to have created more time for themselves to engage in activities which they enjoy. The second type of experience, however, appears more negative, with participants experiencing increased home pressures through having to take on multiple roles within the home which were not present or have increased in comparison to pre-lockdown (e.g., working from home, home education, childcare), or they have had their main source of earning removed. Individuals here express more negative psychological experiences, too, which align to the increased pressures and uncertainty which they are experiencing.

The current research did not examine the effects of caregiver stress and anxiety on those for whom they were caring. However, given the findings indicating that there are two key types of experiences of lockdown, and that much of this appears associated with caring responsibilities, it is sensible to discuss the potential psychological impacts on not only the caregiver but also for those receiving care. During the initial lockdown periods, data collected across international studies indicated, like our findings, that caregivers were experiencing negative affect such as higher levels of stress. For instance, Altieri and Santangelo (2021) found that carers of people with dementia experienced higher levels of depression as a result of lockdown, and that caregiver burden was negatively correlated with resilience and positively correlated with functional dependence. Those who had higher levels of measured resilience were also found to have lower levels of anxiety. These findings present a complex picture of caregiver experiences during lockdown, suggesting that it is not a universal one; similar to our findings, there is nuance in how people cope and experienced living in lockdown, and the extent to which others rely on the caregiver (‘caregiver burden’) and the coping mechanisms (e.g., ‘resilience’) influence the extent of the psychological effects experienced. Within the UK, a large-sample study (*N* = 9737) conducted by Whitley et al. (2021) found similar psychological outcomes within unpaid carers. In their study, unpaid carers of children under 18 years of age reported improvements in their mental health, which was recorded via pre- and in-lockdown comparisons of General Health Questionnaire (GHQ-12) scores. Carers of adult children, on the other hand, and those who reported a greater caregiver burden saw decreases in their mental health, and this was worse for those who had previously received formal help pre-lockdown.

Further to this, in a large sample (*N* = 2988) exploring parent-child mental health in the first UK lockdown, Raw et al. (2021) found that children who experienced elevated symptoms of mental health distress were more likely to also have a parent/caregiver with higher levels of psychological distress. However, the authors highlighted that the picture is not a universal one, and that the experience of lockdown is individual and multi-faceted. This is an important area for further exploration for future research, as care experiences do not exist in a vacuum but are relational in nature.

Similar to the current study’s findings, and taken together, these findings suggest that lockdown experience and its effect on mental health and psychological wellbeing is related to not simply the presence of a caregiving role, but also to the coping mechanisms employed and the resources available to the caregiver in their environment. It is therefore appropriate to suggest that reinstating formal support and increasing formal support available to caregivers and care recipients will be beneficial to psychological wellbeing post-lockdown.

As mentioned at the outset, most research to date has focused on the negatives of lockdown, however, as we find, it might be useful to also explore the potential positives of lockdown. Identifying the positives and who may experience these can be as useful as recognizing what makes a person’s experiences of lockdown negative. Together, this understanding can be used to inform public health policies. In regard to operationalizing the findings from the current paper within health and social care related policy and practice, we must consider these findings in light of others to gain the best possible or most rounded insight into this complex area. For instance, when considering the impact of lockdown on the mental health and wellbeing of children and young people and of those with intellectual or learning disabilities, we see some similarities with the current study’s findings for neurotypical adult populations; although the negative effects of lockdown and the pandemic are heightened in people with intellectual and learning difficulties (Berasategi Sancho et al., 2022). First considering the effects of the pandemic on children and young people and what this means for schooling practices post-lockdown, research by Hornstra et al. (2022) identified that while the experience of students learning from home and having decreased social contact may cause potential learning losses, there are also decreases in motivation and wellbeing. These authors found that these decreases were present regardless of educational level, gender and academic track but were particularly pronounced for across the students with special sensory needs. Similar findings were reported by Berasategi Sancho et al. (2022). Children in this study had increased negative emotions, with increases in crying, feeling nervous, angry and sadder than usual during lockdown and, once again, children with special needs within the sample had lower general wellbeing and experienced more negative emotions. Focus must therefore be given to both emotional wellbeing in addition to the need for academic skill building post-pandemic and particular considerations for the wellbeing of people with special or additional needs being made.

Focusing now on the health and social care experiences of people with learning disabilities, Peacock-Brennan et al. (2021) conducted a service evaluation to help guide planning provision in case of future lockdown situations and to plan for exiting lockdown. As with the previous studies, there are parallels between the current study’s findings and those of Peacock-Brennan et al. (2021). First, these authors reported some positives associated with lockdown experiences, and these highlighted the importance of feeling safe (e.g., messages of safety and consistent guidance), valuing relationships, and keeping busy; which were also key findings of the current research. Participants also reported that missing friends and family in meaningful social contact was a negative. These findings and ours indicate that meaningful contact, social support, and meaningful activities added value during the lockdown period. In terms of public health priorities, emerging from the pandemic, particular support and resource ought to be paid to these key areas – connections with people, whether these are formal or informal caregivers, is clearly highly important, as is meaningful connections and activities. Similar to the recommendation around education, there must therefore be attention and resource allocated to emotional and psych-social wellbeing for people in and post-lockdown. With ongoing resourcing challenges, this meaningful contact could be facilitated via the use of simple technology (e.g., telephone, video-appointments) and operationalized in a routine manner (Peacock-Brennan et al., 2021) to help maintain supportive contact and to act as preventative care indicator.

Consistency of public health messaging and communication around safety was also highlighted in both our and Peacock-Brennan et al.’s (2021) study as important. Based on the balance of our findings and those of others discussed, there ought to be consistent and equitably applied public health messaging and practice which supports not only the physical safety of people’s health, but also considers and supports psychological and social wellbeing.

Furthermore, given the lack of regional differences observed in the current study (see also Dhami et al., 2020), a more unified international approach to public health policies on future lockdown restrictions for regions which are similar may be beneficial. Whilst presenting an international comparison across demographic groupings, the current study cannot fully report on the more nuanced cultural differences and contexts across these two groups of participants as nuanced cultural data were not collected. This may be an important area for further exploration and comparison to best inform more nuanced aspects of public health policies.

Specific comparisons of successful and less successful lockdown approaches taken by governments will no doubt emerge as time progresses and greater access to longitudinal data is available. Informing future approaches to lockdown restrictions based on balancing the variables that achieve both effective infection reduction and which reduce the negative and increase the positive experiences of lockdown on mental health and wellbeing is imperative to reducing both the physical harm caused by infection and the psychological harm caused by restrictions on freedom (see also Bavel et al., 2020).

The findings on shared negative—and positive—experiences of lockdown and the sense of social isolation may also be useful in informing policy and decision making toward provision of support for people who live alone or who are socially isolated outwith pandemic measures due to, for example, mental and physical health issues. The lessons hard-learned by necessity throughout the pandemic restrictions can be used to inform better support for people who are socially isolated and their ongoing health and wellbeing needs. An important consideration is the ethical use and application of findings such as those in the current paper: while we report that lockdown experiences are now wholly negative, stay-at-home restrictive measures such as lockdowns are not normalized aspects of society and, as discussed earlier, there must be balance between the benefits of saving lives through restrictive measures—only applied when absolutely essential—and the emotional, social and psychological wellbeing needs of people and societies. The findings from this paper demonstrate the resilience of people across different regions in response to an unprecedented global phenomenon. That said, the interpretation and discussion of the findings further highlight the need for appropriate planning for physical and mental health support for people in- and post-lockdown, particularly those who are living in isolation.

## Supplementary Materials

The supplementary materials provided are the full survey, data analysis plan, questionnaire, analysis items, response scales, and tables that support the findings of this study (for access see Index of Supplementary Materials below).



MurrayJ.
DhamiM. K.
McClatcheyK.
Weiss-CohenL.
AytonP.
 (2023). Supplementary materials to "Health, wellbeing, and social interaction: An international and demographic analysis of perceived life changes and the positives and negatives of the Covid-19 lockdown"
[Analysis items, response scales, tables]. PsychOpen. 10.23668/psycharchives.12873
PMC1050820737731890

DhamiM. K.
Weiss-CohenL.
AytonP.
MurrayJ.
 (2020). Life in lockdown
[OSF project page with full survey, data analysis plan, questionnaire]. PsychOpen. https://osf.io/8gvmk/


## Data Availability

The current paper reports on the qualitative data collected within the survey “Life in Lockdown.” While the data is not available, the full survey and associated information about the research can be found in the Supplementary Materials.
